# Modulation of mRNA Expression of Monoacylglycerol Lipase, Diacylglycerol Lipase and Cannabinoid Receptor-1 in Mice Experimentally Infected with *T. gondii*

**DOI:** 10.22088/IJMCM.BUMS.10.2.149

**Published:** 2021-09-01

**Authors:** Sahar Rostami-Mansoor, Narges Kalantari, Tahmineh Gorgani-Firouzjaee, Salman Ghaffari, Maryam Ghasemi-Kasman

**Affiliations:** 1 *Cellular and Molecular Biology Research Center, Health Research Institute, Babol University of Medical Sciences, Babol, Iran. *; 2 *Infectious Diseases and Tropical Medicine Research Center, Health Research Institute, Babol University of Medical Sciences, Babol, Iran. *; 3 *Department of Mycology and Parasitology, School of Medicine, Babol University of Medical Sciences, Babol, Iran. *

**Keywords:** Cannabinoid receptor-1, diacylglycerol lipase, endocannabinoid, monoacylglycerol lipase, Toxoplasma gondii

## Abstract

*Toxoplasma gondii*, an obligate intracellular parasite, infects more than 30% of world's population. This parasite is considered to be neurotropic, and has high tropism for the central nervous system, and potentially induces cryptogenic epilepsy by no clear mechanism. The current study aimed to investigate the alteration of the main components of the endocannabinoid signaling systems in *T. gondii*-infected mice. For this purpose, the levels of mRNA expression of monoacylglycerol lipase (MAGL), diacylglycerol lipase (DAGL) and cannabinoid receptor-1 (CB1), were measured by quantitative real time PCR.The mRNA expression level of MAGL was increased by ~ 8-fold in the brains of the *Toxoplasma*-infected group in comparison with non-infected mice (P<0.0001). The mRNA expression of CB1 gene in the brain of the infected mice was ~ 2 times higher than that measured in control group (P<0.01). The mRNA expression level of DAGL remained unchanged in the infected mice. Overall a substantial increase in MAGL and CB1 expression without any changes in DAGL, in the brain of infected mice suggests that *T. gondii* disturbs the endocannabinoid signaling pathways, which are known as neurotransmitter modulators involved in epilepsy.

*Toxoplasma gondii*, an obligate intracellular parasite, infects more than 30% of world's population. This parasite is considered as neurotropic, and has high tropism for the central nervous system (CNS) (1). Previous scientific literature has revealed that both acute and chronic infections by *T. gondii* are associated with neurobehavioral and neurological abnormalities in humans and murine models (1, 2). The influence of *T. gondii *may manifest as different neurological presentations such as epilepsy and seizure (1).

Epilepsy is a common neurological disease that affects over 70 million people worldwide. The main cause of epilepsy is not clear in approximately 60% of patients which is called cryptogenic epilepsy (3, 4). One important causative factor for cryptogenic epilepsy is an infectious disease named toxoplasmosis (5). This infection causes convulsions without any other clinical manife-station in chronic phase, while immunosuppressed status converts chronic toxoplasmosis to acute toxoplasmosis and leads to recurrent convulsions (5). Furthermore, systematic review and meta-analysis studies have shown that toxoplasmosis should be considered as a risk factor for epilepsy (6, 7). However, the molecular mechanism of the interaction between *T. gondii* and epilepsy is not clear. It is plausible that the parasite may influence neurological function through alteration in neurotransmitters, receptors, ion channels, and other central components of brain physiology (8). Modulation in the expression of host genes, and disruption of GABAergic and glutamatergic signaling pathways were observed in *T. gondii*-infected mice (9, 10). The regulation of these neurotransmitters is affected by other molecules such as cannabinoids which plays an important role in the physiological and pathological processes of epilepsy (11). 2-Arachidonoylglycerol (2-AG) is the most abundant endocannabinoid that is produced in the CNS. It is synthesized from diacylglycerols (DAG) by diacylglycerol lipase (DAGL). It has been demonstrated that 2-AG acts as a retrograde messenger in CNS by binding to the cannabinoid receptor-1 (CB1) and is finally degraded by monoacylglycerol lipase (MAGL)(12).

Based on the aforementioned evidence regarding the association between *T. gondii* and epilepsy, and also between epilepsy and endocannabinoids, it was assumed that *T. gondii* may induce epilepsy through disrupting the cannabinoid pathway. Therefore, the current study was conducted to evaluate the changes in gene expression of MAGL, DAGL and CB1 in mice brains, experimentally infected with *T. gondii* RH strain.

## Materials and methods

**Animals**

Twelve 7-week-old male NMR mice were obtained from the laboratory animal center, Babol University of Medical Sciences, Babol, Iran. The mean mice weight was 30 ± 1 g. Animals were housed in groups of four in polypropylene cages with woodchip bedding, access to food and water, controlled temperatures, and 12-hour light/dark cycle. Two mice were used for the first step of the process and the rest of the mice were applied for the main experiments, as described below. All experimental protocols were conducted according to the guidelines of Animal Care and Research Committee of Babol Medical University (Babol, Iran) (IR.MUBABOL.HRI.REC.1398.299).

**Toxoplasmosis induction in mice**

Tachyzoites of *T. gondii* RH strain (Babol University Medical Sciences, Babol, Iran) (13) were retrieved from -80°C and injected intraperitoneally to two mice that were then monitored daily to observe abnormal physical signs such as weakness, immobility, and ataxia. The tachyzoites of T. gondii, RH strain were collected through peritoneal washing, seven days post inclusion Tachyzoites were washed twice using phosphate buffer saline (PBS) (pH 7.5) and counted by hemocytometer. A suspension containing 10^6^ tachyzoites was injected intraperitoneally to each mouse in the infected group (n = 5). Mice in control group received sterile PBS (n = 5). All animals were monitored daily for clinical manifestations of acute toxoplasmosis. Four days after treatment and following clinical signs of toxoplasmosis, animals were anesthetized by a mixture of ketamin and xylazine. The whole brains were harvested and rinsed with PBS and crushed on ice into tiny portions. 100 mg of the crushed brain was suspended in TriPure Isolation Reagent. The collected samples were kept at-80°C for further use.

**DNA extraction and polymerase chain reaction (PCR) amplification**

Total DNA was extracted from 30 mg brain tissue using a commercial kit (PCRBIO Rapid extract PCR kit, UK) according to the manufacturer’s instructions. The extracted DNA was kept at -20°C until used. PCR was carried out for all extracted DNA using specific primers to amplify a 321 bp fragment of the B1 gene of *T. gondii*. The forward and reverse primers of the B1 gene were selected according to Rahumatullah *et al*. (2012) (14) (Table 1). The PCR amplification was performed in a total volume of 25 μL with the following thermal program: denaturation at 94°C for 5 min, 40 cycles of denaturation at 94 °C for 30 s, annealing at 59°C for 15 s, and extension at 72°C for 30 s and a final extension step at 72°C for 7 min. The PCR products were electrophoresed on a 2% agarose gel, and the bands were visualized under UV light and photographed using a gel documentation system (Vilber Lourmat, France).

**Quantitative real time- PCR**

Total RNA was isolated from the brain using TriPure Isolation Reagent according to the manufacturer’s instruction (Roche, Germany). The RNA pellet was dissolved in 70 mL DEPC–water, and kept at -80°C. Complementary DNA (cDNA) was prepared using random hexamer primers by reverse transcriptase kit (Yektatajhizazma, Tehran, Iran). The genes were amplified using specific primers for MAG1, DAGL and CB1 genes (Table1) and SYBR Green master mix (Amplicon, Odense, Denmark) on Rotor gene PCR machine (Qiagen, Germany). q-RT-PCR reactions were repeated two times in triplicate under following conditions: initial denaturation at 95°C for 5 min, followed by 40 cycles of denaturation at 95°C for 10 s, annealing at 59°C for 30 s, and extension at 72°C for 30 s. Target gene expression was normalized against housekeeping gene hypoxanthine phosphoribosyl transferase (*Hprt*) expression. The fold changes between groups were evaluated using relative quantification (2^−ΔΔCt^) method.

**Statistical analysis**

The data was statistically analyzed by Graph Pad Prism software version 6.01. All data are presented as mean ± SEM. Unpaired t-test was applied for the comparison of gene expression between the infected group and control group, and results were considered significant for P < 0.05.

**Table1:** The sequences of primer pairs for genes used for PCR amplification and quantitative real-time PCR

**Target gene**	**Primer sequence (5 ′ → 3′)**	**Amplicon size (bp)**
**CB1**	F: AGACCTATACCCACACCCCT R: AAGCTAGCCACCCTCATCT	226
**MAGL**	F: ACTAGGAGTTGCTTGCCAGT R: GCTTGGGTTTCACTGCTTCA	215
**DAGL**	F: CTGTGGTTCTGGGCAAAGAC R: CGAAAGGGCGATGGTCAAAT	233
**HPRT**	F: ATTATGCCGAGGAT TTGGA R: ACTTATAGCCCCCCTTGA	141
**B1**	F: ATAGGTTGCAGTCACTGACG R: CTCCTCTTCGCGAAACCTCA	321

## Results

**Toxoplasmosis confirmation in infected mice**

**Fig.1:**
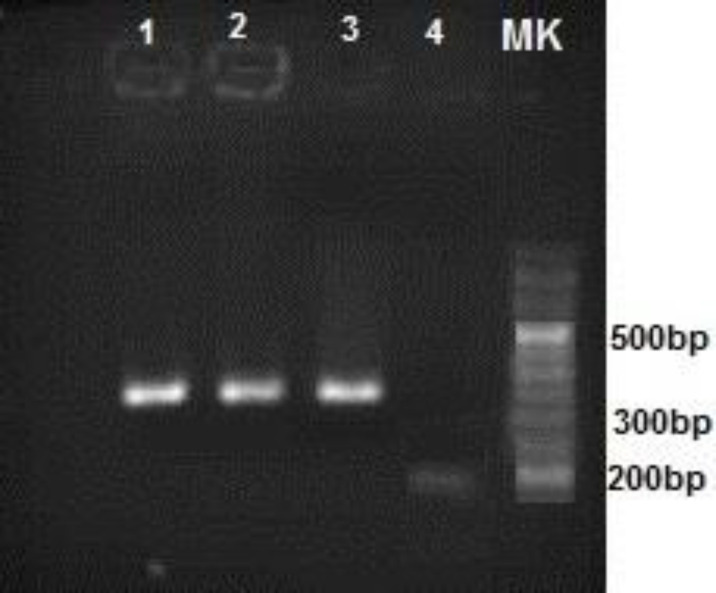
**PCR amplification of the B1 gene of** ***T. gondii*** **genome from the brains of infected mice**. Lane 1 and 2: positive samples; lane 3: positive control; lane 4: negative control; MK: 50 bp marker.The amplicon size was 321 bp

Clinical symptoms related to toxoplasmosis such as weakness, immobility and ataxia were observed in the inoculated mice two days post infection. No symptoms were seen in control group. The presence of *T. gondii* DNA in the brain sample of infected mice was confirmed by PCR analysis of the B1 gene of *T. gondii* and observation of a 321 bp band on agarose gel (Figure 1).

**Changes of CB1 expression at mRNA level in Toxoplasma-infected mice**

**Fig.2:**
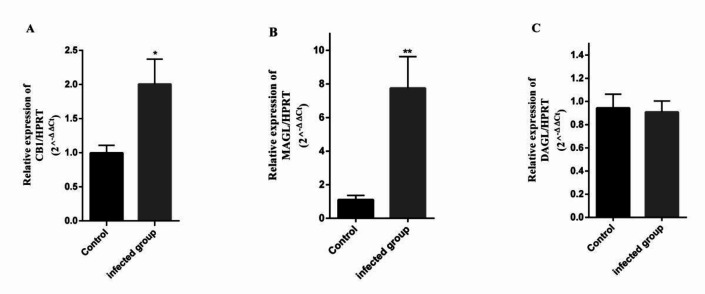
**Comparison of CB1, MAGL and DAGL expression at mRNA level in the brain of** ***T. gondii*** **infected mice.** A) CB1, B) MAGL, C) DAGL mRNA levels in the brain of mice infected by RH strain of *T. gondii* compared to the control group. The Ct values were normalized using *Hprt* as the endogenous control. Data are presented as mean ± SEM. *P < 0.01; **P < 0.0001 vs. control group. (n = 5 per group).

As shown in Figure 2, the expression of CB1 gene in the brain of infected mice was~ 2 times higher than that measured in the control group (P<0.01), while no changes were observed in the expression level of DAGL as a synthetic enzyme in comparison with the control group. The expression of mRNA specific to the degrading enzyme, MAGL, in the brain of the infected group was ~ 8 folds higher in comparison with the control group (P <0.0001*)* (Figure 2).

## Discussion

*T. gondii* has been considered as a potential cause of cryptogenic epilepsy in pediatric groups and immuno-compromised people (15). Several studies have tried to demonstrate the pathology of epilepsy induction by *T. gondii* in human and animal models. Some studies have illustrated that GABAergic and glutamatergic disruption occurred in toxoplasmosis-induced seizures (10, 16). On the other hand, cannabinoids exert various effects as pro- or anti-convulsive agents in animal models such as fever-induced seizures and pilocarpine model of temporal lobe epilepsy (17).

In the present study, we showed that *T. gondii* disrupts MAGL and CB1 genes expression. It is well known that *T. gondii* is capable of interfering with the hosts’ molecular processes, through either direct interaction or indirect mechanisms (18). *Toxoplasma* releases parasite-encoded effector proteins that change the biological system in regions with no tachyzoites or bradyzoites. These changes may occur anywhere in the brain (18). On the other hand, the overexpression of MAGL which has a degrading effect on 2-AG, without any significant change in DAGL expression that was observed in the present study, suggests that the disruption of the cannabinoids system may play a role in the induction of epilepsy. Our results have been supported by a very recent study which found that seizure threshold was increased in *Toxoplasma*-infected mice treated with JZL184 (the MAGL inhibitor) (19). Moreover, other studies have shown that the treatment of temporal lobe epilepsy, fully kindled and PTZ-induced seizure by MAGL antagonist decreased seizure in animal (20, 21). Additionally, arachidonic acid which is produced from 2-AG hydrolysis by MAGL, enhances the risk of epilepsy and other neurodegenerative disorders (22).

We were also able to demonstrate that the expression of CB1 in the brain of infected mice was approximately 2 folds higher than the control group. There are several studies that have revealed that central or peripheral inflammation induces CB1 expression in the CNS to suppress the excessive inflammation (23). Acute toxoplasmosis corres-ponds to neuroinflammation in the brain of mice (24), therefore it is suggested that increased CB1 expression may be a compensatory response to inflammatory cytokine and chemokine decline in neural dysfunction. Furthermore, CB1 agonists are putative anticonvulsant drugs as described by Ghanbari et al. (2020), which reported that the adminstration of arachidonyl-2'-chloroethylamide (ACEA) inhibited the proconvulsant effect of toxoplasmosis in mice (19).

The amount of MAGL, DAGL and CB1 at protein level, and the expression level of 2-AG were not evaluated in the present study.

In conclusion, this is the first study evaluating the potential changes in endocannabinoid systems in Toxoplasma infected mice. We have shown that a disturbance in the endocannabinoid system following toxoplasmosis may have a role in epilepsy. It seems that the overexpression of MAGL is one of the important mechanisms in inducing epilepsy or even other neuropathological conditions in cases with infections with *T. gondii*. More studies should be conducted to reveal the alteration of the endocannabinoid system at protein level, which are involved in signaling pathways. 
